# Biomedical Applications of Titanium Alloys: A Comprehensive Review

**DOI:** 10.3390/ma17010114

**Published:** 2023-12-25

**Authors:** Elia Marin, Alex Lanzutti

**Affiliations:** 1 Ceramic Physics Laboratory, Kyoto Institute of Technology, Sakyo-ku, Kyoto 606-8585, Japan; 2 Department of Dental Medicine, Graduate School of Medical Science, Kyoto Prefectural University of Medicine, Kamigyo-ku, Kyoto 602-8566, Japan; 3 Department Polytechnic of Engineering and Architecture, University of Udine, 33100 Udine, Italy; 4 Biomedical Research Center, Kyoto Institute of Technology, Sakyo-ku, Kyoto 606-8585, Japan

**Keywords:** titanium alloys, biocompatibility, orthopedics, dental implants, cardiovascular devices, 3D printing, osseointegration

## Abstract

Titanium alloys have emerged as the most successful metallic material to ever be applied in the field of biomedical engineering. This comprehensive review covers the history of titanium in medicine, the properties of titanium and its alloys, the production technologies used to produce biomedical implants, and the most common uses for titanium and its alloys, ranging from orthopedic implants to dental prosthetics and cardiovascular devices. At the core of this success lies the combination of machinability, mechanical strength, biocompatibility, and corrosion resistance. This unique combination of useful traits has positioned titanium alloys as an indispensable material for biomedical engineering applications, enabling safer, more durable, and more efficient treatments for patients affected by various kinds of pathologies. This review takes an in-depth journey into the inherent properties that define titanium alloys and which of them are advantageous for biomedical use. It explores their production techniques and the fabrication methodologies that are utilized to machine them into their final shape. The biomedical applications of titanium alloys are then categorized and described in detail, focusing on which specific advantages titanium alloys are present when compared to other materials. This review not only captures the current state of the art, but also explores the future possibilities and limitations of titanium alloys applied in the biomedical field.

## 1. Introduction

In recent decades, the intersection of materials science and biomedical engineering has yielded remarkable advancements in biomedical engineering. At the forefront of this convergence stands titanium, an elemental metal that, when properly alloyed, has proven itself to be a game-changing biomaterial [1]. Renowned for its unique blend of mechanical strength [2], biocompatibility [3], corrosion resistance [4], and the ability to promote integrative tissue interactions [5], titanium alloys have emerged as the most successful material in biomedical applications, in particular in the fields of orthopedics and dentistry [6].

The present comprehensive review tries to navigate the intricate landscape of titanium alloys’ biomedical applications—a journey that traverses numerous medical disciplines, from orthopedics [7] to dentistry [8] and even cardiovascular care [9]. With an emphasis on elucidating the core attributes that underpin their exceptional versatility, we delve into the mechanisms that are responsible for the success of titanium alloys, but also the limitations.

The story of titanium alloys’ foray into the medical world is closely intertwined with the groundbreaking work of Dr. Per-Ingvar Brånemark [10,11,12]. His pioneering research on osseointegration—a term he coined to describe the direct structural and functional connection between living bone and the surface of an implant—ushered in a new era of medical possibilities. The realization that titanium could serve as a “scaffold” to support—if not stimulate—bone tissue adhesion, growth, and integration led to a revolution in orthopedic and dental implantology. The impact of Brånemark’s contributions continues to reverberate, as his insights laid the foundation for the use of titanium and its alloys in modern medical applications and, in the last decades, no other structural metal was ever able to achieve the combination of performances shown by titanium.

In orthopedics, titanium is the most common choice for components that undergo heavy, cyclic mechanical solicitation, in particular for stems and cups in articulations such as shoulder, hip, knee, and ankle, for which polymeric materials would not reach the necessary strength. Apart from strength, titanium implants provide good secondary fixation as they undergo osteointegration over time [13], and the process can be further accelerated using cellular solid morphologies [14]. On the other hand, despite the scientific interest and the numerous attempted solutions [15], titanium has been shown to be unsuitable for components subjected to wear, such as in the case of femoral heads [16] in hip arthroplasty or femoral components [17] in knee arthroplasty.

In the field of dentistry, titanium has found application for dental posts, screws, abutments, braces, instruments, and also temporary devices. Compared to orthopedics, devices used in dentistry and orthodontics are under lower mechanical solicitations, but the oral cavity is a much harsher environment from the chemical and biological point of view. Constant changes in pH [18], release of aggressive chemical species from foods and beverages [19], presence of tartar/plaque [20], and proliferation of pathogens such as bacteria [21] can drastically reduce the chances of clinical success and the expected life span of biomaterials. In most cases, the chemical inertness of titanium has proven to be up to the task, but concerns are periodically raised about the potential release of harmful alloying elements [22], and allergic reactions have also been reported, in particular for titanium–nickel alloys [23].

Like orthopedics appliances, trauma devices such as screws, plates, and intramedullary nails are also subjected to intense solicitations, to the point that it is not unusual for surgeons to prefer the more resistant stainless steel over titanium [24]. Moreover, unlike most components used in arthroplasty, trauma devices are often removed after the healing process has been completed [25,26], and a perfectly osseointegrated titanium device would prove difficult to remove without damaging the surrounding tissue [27,28].

Despite spinal devices being technically a subset of orthopedic implants, they possess unique characteristics and, for the scope of this review, will be considered a separate category. Spinal implants that can make use of titanium or titanium alloys are pedicle screws, rods, inter-body cages, artificial discs, and all the hooks and wires that are used in the correction of spinal deformities such as scoliosis, so depending on the application, the device can be articulating (artificial discs) or locking (inter-body cages) bones into position, behaving either like an arthroprosthetic or a trauma device. Despite being, in most cases, subject to relatively low mechanical stresses, spinal implants face complex anatomy [29] with a great degree of variability among patients and can cause damage to the nearby nerves [30] and blood vessels and breakage is the most common type of failure in devices such as pedicle screws [31]. Moreover, the stiffness of titanium can delay the healing processes [32] or even cause unexpected bone fractures.

While titanium alloys’ preeminence in the field of orthopedics and dentistry has long been established, the combination of mechanical strength and biocompatibility promoted the use of titanium and titanium alloys in other fields of medicine, such as in cardiovascular devices. Titanium can be used as the main component of stents, heart valves, and vascular grafts, as well as parts of implantable defibrillators, pacemaker cases, and implantable sensors. Apart from biocompatibility, the most important requisite for cardiovascular implants is to be reliable over time, meaning that they are supposed to function without revision for long time spans. The revision of cardiovascular implants carries additional risks and, unlike most arthroprosthetic or dental devices, the mechanical malfunction of a cardiovascular device is life-threatening [33].

**Figure 1:**
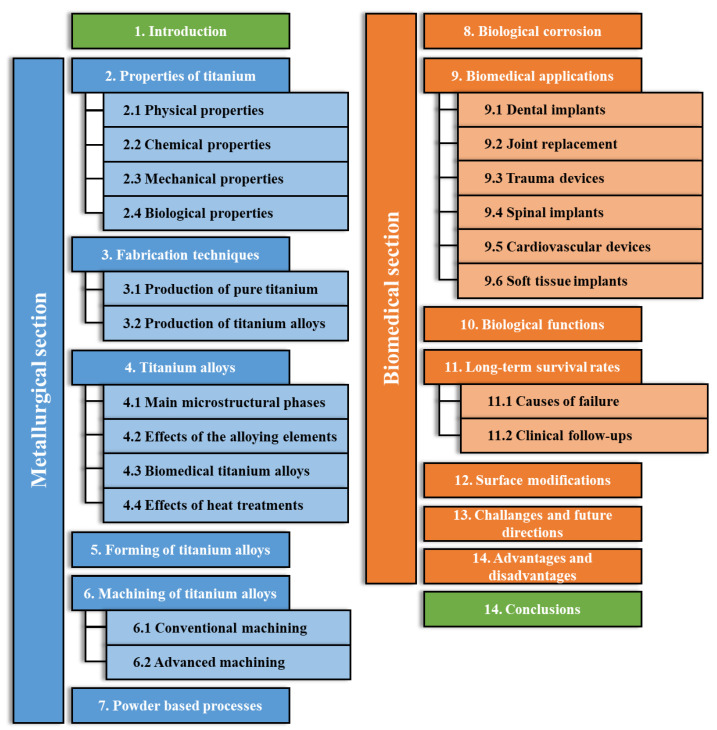
Summary diagram of the contents of this review, with the numbers and titles of sections and sub-sections.

The main contents of this review, as well as the titles of sections and sub-sections, are summarized in Figure 1.

## 2. Properties of Titanium

Titanium is a chemical element with the symbol “Ti” and atomic number 22, nowadays recognized for its unique qualities that find practical use in various industries, from aerospace to automotive, military, sports, and even jewelry.

### 2.1. Physical Properties

Titanium undergoes a phase transformation from a hexagonal close-packed (HCP) crystal structure to a body-centered cubic (BCC) structure as a function of temperature. At room temperature, commercially pure titanium primarily exists in the α-phase, which has an HCP arrangement. As the temperature rises above approximately 883 °C (1621 °F), it transforms into the β-phase, characterized by the BCC arrangement [34]. This phase transition has an impact on various properties of the material, such as ductility and strength.

Titanium has a relatively low density of around 4.5 g/cm³, which is approximately half the density of steel or cobalt alloys. This low density contributes to its lightweight nature, making it highly desirable for applications where weight reduction is essential, such as in aerospace and medical implants. The low density is a key factor in reducing the overall load and movement inertia on the human body when titanium implants are used.

Titanium exhibits favorable thermal properties [35], including a high melting point of approximately 1668 °C (3034 °F). This high melting point enables the material to withstand elevated temperatures during manufacturing processes without losing its structural integrity. Additionally, titanium has a low thermal expansion coefficient, which means it expands and contracts minimally when subjected to temperature changes. This property is advantageous for applications where dimensional stability is crucial, such as in precision medical devices.

Titanium is a relatively poor conductor of electricity compared to materials like copper or aluminum, making it useful for applications where electrical insulation is desired [35]. In certain medical applications, such as implantable medical devices, the low electrical conductivity of titanium can be advantageous to prevent unwanted electrical interactions with the body’s tissues.

### 2.2. Chemical Properties

Thanks to its affinity towards oxygen, when exposed to oxidizing environments titanium spontaneously forms a superficial layer of protective oxide. This oxide layer is essential for the corrosion resistance of titanium and plays a crucial role in its biocompatibility and other applications, in particular at high temperature. Titanium oxide has two different crystalline forms, anatase and rutile, and their formation depends on the environmental conditions. Similarly, the thickness of the native oxide layer on titanium can vary depending on factors such as exposure conditions and the specific alloy composition, generally ranging from a few nanometers to about 10–30, and alloys exposed to humid air at room temperature have shown to form prevalently composite layers of titanium (IV), titanium (III), and titanium (II) oxides [36].

In biomedical applications, the presence of the native oxide layers plays a crucial role in biocompatibility [37], as it forms a barrier between the biological environment and the “reactive” metal underneath. The layer acts as a protection both against corrosion, as the biological environment is particularly aggressive towards metals, and against adverse reactions such as severe inflammations.

### 2.3. Mechanical Properties

Titanium alloys exhibit impressive mechanical strength and stiffness, providing the required load-bearing capacity for applications like orthopedic implants and dental prosthetics. The specific mechanical properties can be tailored by alloying with other elements, with some alloys reaching ultimate loads comparable with those of technical steels. Like steels, titanium alloys possess a fatigue limit [38] and display excellent fatigue resistance [39], making them suitable for long-term use in dynamic environments, such as orthopedic implants subjected to cyclic loading. Moreover, titanium alloys have a relatively low modulus of elasticity compared to other metallic materials applied in the biomedical field, which helps reduce the stress shielding effect, minimizing the loss of bone density around implants.

### 2.4. Biological Properties

Despite acting as a physical barrier between the metal and the biological environment, the native layer of titanium oxide formed on the surface of titanium and its alloys is not completely inert. As observed by Dr. Brånemark, bone tissue can adhere and grow on the surface of titanium alloys, to the point that titanium-based devices can be completely osseointegrated over time [40,41,42]. Titanium’s ability to integrate with bone, known as a key biomedical property, and various types of biomedical devices, such as scaffolds, makes use of this property to accelerate bone tissue regeneration or to improve adhesion and stabilization. Another property associated with the presence of native oxides is that titanium is considered hypoallergenic, as it rarely triggers allergic reactions in patients. This is especially important for medical devices in prolonged contact with the body. Moreover, titanium and its alloys are generally considered non-toxic and they supposedly do not release harmful substances into the body, ensuring patient safety over extended periods. Some concerns have been raised on the potential toxicity of a few, specific alloys, such as titanium grade 5 [43] or Nitinol [44], due to the presence of aluminum/vanadium and nickel, respectively.

## 3. Fabrication Techniques

### 3.1. Production of Pure Titanium

Titanium is primarily produced using methods that are specific to its unique characteristics. The two historically more relevant processes for producing commercially pure titanium are the Kroll process and the Hunter process:

*Kroll Process* [45]: The Kroll process is a primary method for producing titanium metal from its ores. In this process, titanium tetrachloride (TiCl_4_) is obtained by reacting titanium ores (typically ilmenite) with chlorine gas. The TiCl_4_ is then reduced to metallic titanium using magnesium metal in a high-temperature reactor. The reaction forms magnesium chloride as a byproduct. The process involves multiple stages, including chlorination of the ore, condensation of TiCl_4_, reduction of TiCl_4_ by magnesium, and separation of titanium sponge from magnesium chloride. The produced titanium sponge contains impurities and must undergo further processing steps to purify and refine the metal. Despite its complexity, the Kroll process remains a fundamental method for industrial titanium production, especially for aerospace, medical, and industrial applications.

*Hunter Process* [46]: The Hunter process is an alternative method for producing titanium metal from its ores. In this process, titanium tetrachloride (TiCl_4_) is also obtained by chlorination of titanium ores like ilmenite. However, instead of using magnesium for reduction, the Hunter process employs sodium or a sodium–potassium alloy as the reducing agent. The reduction takes place at a lower temperature compared to the Kroll process. A sodium or sodium–potassium alloy reduces TiCl_4_ to form metallic titanium and sodium or potassium chloride as byproducts. The resulting titanium sponge is then processed further to remove impurities and refine the metal. The Hunter process offers advantages like lower operating temperatures and reduced energy consumption compared to the Kroll process. However, it is less commonly used in industrial titanium production.

### 3.2. Production of Titanium Alloys

To produce titanium alloys starting from commercially pure titanium, particular care has to be given to the prevention of oxidation. For this reason, only a few technologies are commonly used:

*Vacuum Arc Remelting* (*VAR*) [47]: VAR is a commonly used technique for melting and refining titanium alloys. It involves melting a consumable electrode under vacuum conditions using an electric arc. This process helps to reduce impurities and control the alloy’s composition.

*Plasma Arc Melting* (*PAM*) [48]: PAM is another vacuum-based melting technique that employs a high-energy plasma arc to melt the material. It offers better control over composition and is suitable for producing specialty alloys.

*Induction Melting* [49]: Induction melting is used to produce small quantities of titanium alloys. It involves using electromagnetic induction to melt the material within a crucible in a controlled atmosphere.

*Powder Metallurgy* [50]: Powder metallurgy is mainly used as a near-shape production technique for titanium components, but by mixing powders with different chemical compositions, it is possible to use it as an alloying technique as well.

It is important to note that these production techniques are specialized and require stringent process control due to titanium’s reactivity and sensitivity to impurities. The choice of production method depends on factors such as the desired alloy composition, the quantity of material needed, and the specific properties required for the end application.

The formation of the undesirable alpha case [51], a hard and brittle layer of alpha-phase titanium, poses challenges during production processes involving high temperatures and reactive environments [52]. The alpha case can weaken mechanical properties [51] and induce implant failure. To counteract this, careful control of processing parameters and the utilization of protective environments are essential.

Moreover, the biocompatibility of titanium alloys depends on element concentrations. Elements such as aluminum and vanadium in excess can trigger toxic effects, thus stringent controls on composition are necessary [53,54,55]. For instance, Grade 5 titanium (Ti-6Al-4V) used in orthopedic implants and dental prosthetics, requires careful alloying to avoid cytotoxicity.

## 4. Titanium Alloys

### 4.1. Main Microstructural Phases

Most titanium utilized for the production of biomedical devices is alloyed with other elements, and even the commercially pure titanium grades actually contain small amounts of trace elements that have a clear influence on their final properties.

As discussed in Section 2, titanium alloys exhibit mainly two primary crystalline structures: alpha (α) and beta (β), where the α-phase has a hexagonal close-packed structure and the β-phase a body-centered cubic (BCC) structure. The partition between these phases significantly influences the material’s mechanical properties. Pure titanium has a transition temperature, known as the beta transus temperature, above which it transforms from the α-phase to the β-phase, but the transformation is reversible so commercially pure titanium grades do not have stable residual β-phase when they are cooled down from temperatures above the beta transus. Before discussing the effects of the alloying elements on the stability of the α- and β-phases, it is important to consider that titanium alloys, as basically all other metals, can also form several both stable and metastable phases. A comprehensive list of titanium alloy phases, along with their symbols and space groups, is listed in Table 1.

### 4.2. Effects of the Alloying Elements

**Figure 2:**
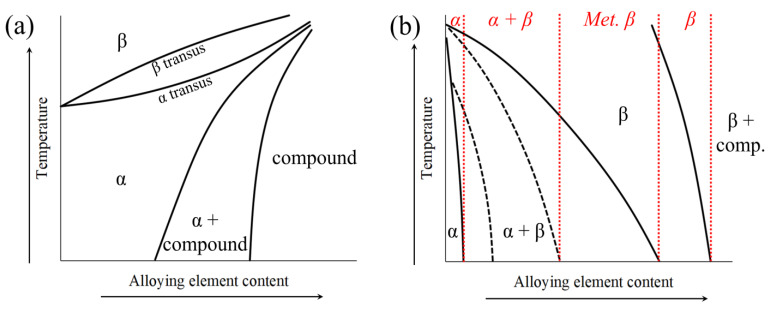

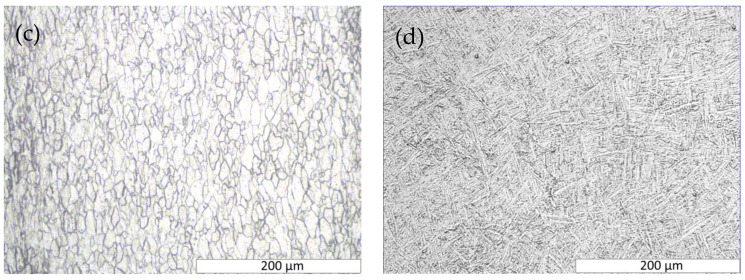
Effects of (**a**) alpha and (**b**) beta stabilizers on the microstructure of titanium alloys. The dotted black lines represent the region of metastability of α-β alloys, the red dotted lines represent the expected microstructure at room temperature, (**c**) an example of a typical commercially pure α-titanium microstructure, and (**d**) an example of a typical annealed Ti-6Al-4V α-β alloy microstructure.

Alloying elements play a pivotal role in tailoring the properties of titanium alloys. Traditionally, titanium-alloying elements are divided into two groups, depending on the phase they tend to stabilize. Alloying with elements like aluminum, tin, and zirconium stabilizes the α-phase, enhancing strength and hardness, and increasing the beta transition temperature [62]. Elements like molybdenum, tungsten, chromium, iron, silicon, and copper stabilize the β-phase, improving ductility and high-temperature performances, and also decreasing the beta transus temperature [56]. Vanadium and niobium can act as an α- or β-stabilizer, depending on the composition of the alloy [63]. Additionally, light elements such as oxygen and nitrogen also have an α-stabilizing effect and a neutral effect on beta transus temperature. The role of alpha and beta stabilizers in the control of the microstructure of titanium alloys is summarized in Figure 2.

The combination of these alloying elements allows for the creation of alloys with a wide range of mechanical properties, biocompatibility, and corrosion resistance. Conventionally, titanium alloys are divided into five categories, depending on their chemical composition and expected microstructure in service. The five categories are:

*Alpha Alloys* [62]: Primarily α-phase structures. This category comprises both unalloyed titanium and alloys containing α-stabilizers, such as aluminum and tin that are prevalently used for aerospace applications (Figure 2c).

*Near Alpha Alloys* [64]: Predominantly α-phase with limited β-stabilizers. Balance of strength and formability.

*Alpha-Beta Alloys* [65]: Balance of α- and β-phases, offering a combination of strength, ductility, and heat resistance, commonly used in a wide range of applications (Figure 2d).

*Near Beta Alloys* (*or Beta metastable*) [66]: Predominantly β-phases with limited α-stabilizers. When compared to α-β alloys, they sacrifice mechanical strength to improve the ductility.

*Beta Alloys* [67]: Predominantly β-phase alloys with elements like vanadium and molybdenum.

There is another titanium-based structure that is technically considered to be a nickel–titanium intermetallic. For the sake of this review, Nitinol will be considered a titanium alloy, despite containing about 55% nickel. This choice is mainly due to the large number of important biomedical applications and its availability on the market [68]. This material has been added to complete the panorama of Ti-based alloys used in biomedical applications. However, being an intermetallic and not technically an alloy, it will not be discussed in further detail in this review.

### 4.3. Biomedical Titanium Alloys

**Figure 3:**
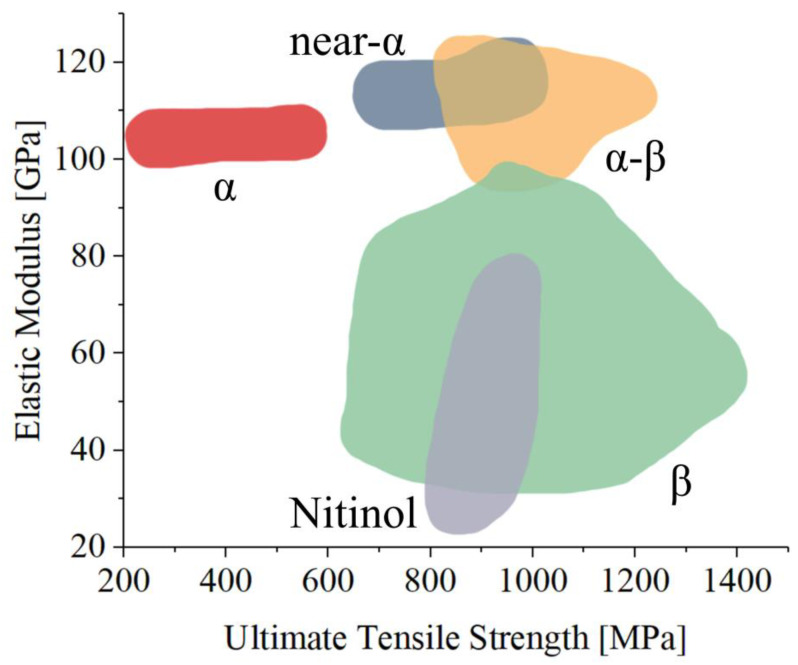
Relationship between ultimate strength and elastic modulus for a selection of alloys commonly applied in the biomedical field, grouped by chemical composition.

The main alloys applied in the biomedical field, along with their microstructures and applications, are listed in Table 2, while the relationship between their elastic modulus and their ultimate strength is presented in Figure 3.

Despite the wide range of possible alloys, commercially pure titanium grade 2 and Ti-6Al-4V grade 5 are used in more than 95% of all titanium biomedical devices. Also widely used in the biomedical field are ELI (Extra-Low Interstitials) alloys, whose main chemical composition is similar to that of the above alloys, but with very low levels of interstitial elements. A reduction in the levels of interstitial elements (O, N, H, and B) in the alloy has a beneficial effect in increasing the ductility and fracture toughness of the material [118].

### 4.4. Effects of Heat Treatments

Like all other alloys, heat treatments can be utilized to control the microstructure and properties of titanium alloys, but the low thermal conduction of titanium makes microstructure control more challenging when compared to other high-performance alloys such as steel. The lack of microstructural control is more evident in thick components where the differences in cooling rates between the core and surface are affected by the above property.

Another important weakness in the heat treatment of Ti alloys is their reactivity with many gases such as O and N, which tend to promote the formation of the detrimental alpha case layer on the outer surface. To avoid this problem, heat treatments are usually carried out under vacuum and the fast-cooling rates are usually achieved by gas cooling with inert gases. This also highlights that the Ti alloys are sensitive to cooling rates during quenching [119,120].

No strengthening heat treatment can be performed on α- and α-near titanium alloys. Both types of alloys can be stress-relieved and annealed (also recrystallization treatment), but high strength cannot be developed in these alloys by any type of heat treatment, as the alloy consists of only one stable phase.

In the case of commercially pure titanium, the trace elements play a critical role in the mechanical performance, and both Oxygen [121] and Iron [122] contents are usually close to the maximum allowed amount to increase the strength of the material. Usually, the O content is lowered to increase the toughness of the material [123].

The three most common heat treatments applied to titanium alloys are [124,125,126]:

*Stress-relieving*: Mainly utilized to reduce undesirable residual stresses that may result during material processing, usually performed at temperatures between 595 and 705 °C (1100 and 1300 °F) for a period of one to two hours, for alpha and Alpha–Beta alloys, and at 700–800 °C with shorter times, for beta alloys. Cooling is usually performed in air. Stress relieving does not alter the overall microstructure or phase distribution. The main effect is to improve the material’s mechanical properties that are sensitive to residual stresses (e.g., fatigue).
*Annealing*: Annealing titanium and its alloys primarily aims to enhance fracture toughness, room temperature ductility, dimensional and thermal stability, as well as creep resistance. As it maximizes some of the most important technological properties, many titanium alloys are placed in service in their annealed state. There are two common types of annealing for titanium alloys that are relevant for biomedical applications, and the most suitable treatment depends on both the chemical composition and the scope:
  − Recrystallization annealing: By heating the alloy up to the upper end of the α-β range (but still below the β-transus), recrystallization annealing helps refine the grain structure of titanium alloys. It promotes the formation of new, smaller grains with a lower dislocation density, which enhances mechanical properties such as strength and ductility. It also contributes to eliminating cold work effects and residual stresses.
  − β-annealing: It involves heating the material within the beta-phase region (but usually as close as possible to the β-transus) to enhance its mechanical properties. Held at a specific temperature range, this treatment facilitates the dissolution of unwanted phases, reduces residual stresses, and promotes the formation of a uniform microstructure.
  − Duplex annealing: Duplex annealing of titanium alloys entails a two-step process. The alloy is initially heated to the beta-phase region for homogenization, eliminating chemical inhomogeneities. Subsequently, a second annealing in the alpha-beta region refines the microstructure, improves mechanical properties, and minimizes residual stresses. Duplex annealing is commonly applied to α-β titanium alloys, particularly those used in aerospace and high-performance applications.
*Solution treating and aging*: In solution treating, the alloy is heated to a high temperature within the beta-phase range to dissolve alloying elements and achieve a homogeneous solid solution. Rapid quenching locks in this solid solution. Aging, the second step, involves reheating the alloy to a lower temperature to encourage precipitation of fine particles. These particles contribute to strengthening and refining the microstructure. The balance between the alpha and beta phases, achieved through proper aging, enhances mechanical properties, such as strength, hardness, and fatigue resistance. The specific temperatures and times for these processes depend on the alloy’s composition and intended application.

Biomedical titanium alloys make use of most of these heat treatments, depending on the production technique, the chemical composition, and the intended application. Forged components, such as femoral stems, usually require a stress-relieving treatment before they can be machined and finished. Nitinol, on the other hand, is usually annealed at high temperature to dissolve precipitates and impurities.

## 5. Forming of Titanium Alloys

Ti alloys can be purchased conventionally as cast or after some plastic deformation (hot or cold working) to shape the component and control the microstructure. The melting process is usually carried out by induction or arc melting under a protective atmosphere to avoid oxidation of the molten bath. The highest quality molten baths are obtained using vacuum processes (VIM or VAR). The material is then cast in water-cooled copper molds to produce ingots, which are then machined to their final shape. Cast materials are not only used for components with complex shapes, such as femoral stems [127,128], but also on more simple devices such as titanium posts for dental applications [129,130,131]. In this case, the liquid metal processed by the above techniques is poured into a mold with a geometry close to the final shape of the component and then sometimes centrifuged to improve uniformity and mechanical properties [132]. The microstructure of the material is related to the solidification conditions and the alloy content.

Ti-alloys can also be formed by intense plastic deformation at high temperatures [133,134,135]. The raw material is usually a sheet or preformed material. Conventional forming consists of forging the material at temperatures in the range of 800–1000 °C. In this case, during the forming process, the most important parameters for forging the material are temperature and strain rate. It is important to limit the gas interaction of the forged material in order to avoid the formation of an alpha case layer. On the other hand, the stamping process is often used to obtain components produced by deforming the material at warm temperatures (300–400 °C) by sheet deformation. By increasing the temperatures and controlling the strain rates, it is also possible to reach a superplastic regime, which is useful for the production of very complex shapes where triaxial deformation is required. Most of the emerging techniques in the biomedical field are related to severe plastic deformation, which results in a material with a significant increase in mechanical properties due to the severe grain refinement that the material undergoes. These processes are equal channel extrusion [136], accumulative roll bonding [137], multidirectional forging [138], and twinning extrusion [139]. The process temperature to obtain a micrograin structure is in the range of 400–600 °C.

## 6. Machining of Titanium Alloys

### 6.1. Conventional Machining

The production of biomedical titanium components involves a range of techniques tailored to meet specific application requirements. For orthopedic and dental implants, the most common production method is precision machining [140], which offers precise geometric control and surface finish. However, machining can introduce residual stresses and potential surface damage, affecting mechanical integrity and implant performance [141,142]. Thus, post-machining treatments are crucial to relieve stresses and restore material properties.

In more complex cases, for example for the production of cellular solids such as scaffolds for tissue regeneration, additive manufacturing (3D printing) has gained prominence, enabling the creation of complex, patient-specific implants with porous structures to promote osseointegration. The effects of 3D printing on the microstructure [143,144,145] and the consequent post-processing treatments [146,147] will be discussed in the next chapter.

Machining titanium alloys can be challenging due to their excellent combination of strength, low thermal conductivity, and chemical reactivity. However, several machining techniques are employed to shape and finish titanium alloy components for various applications. In addition to conventional machining techniques like turning, milling, drilling, and grinding, titanium is frequently processed using more advanced technological methods, such as electrical discharge machining, abrasive water jet cutting, laser machining, and ultrasonic machining. Given that these techniques are less common, a brief description of each is provided in the next paragraph, to benefit the reader.

### 6.2. Advanced Machining

*Electrical Discharge Machining* (*EDM*) [148,149]: Electrical Discharge Machining (EDM) is an advanced metal removal process used to shape intricate and complex geometries in titanium alloys and other materials that are electrically conductive. In the context of titanium alloys, EDM offers a unique advantage due to their inherent hardness and challenging machinability. In EDM, a precisely controlled electrical discharge, or spark, is generated between a tool electrode and the workpiece submerged in dielectric fluid. This controlled spark erosion method selectively removes material from the workpiece without direct physical contact. The dielectric fluid acts as a coolant and insulator, flushing away the eroded particles. For titanium alloys, which possess high melting points and low thermal conductivity, EDM is particularly useful. The process can achieve precision cuts without inducing thermal damage or distortion, which is common with traditional machining methods. EDM is well suited for intricate shapes, thin sections, and hardened surfaces, which are characteristic of titanium components used in aerospace, medical implants, and more. However, EDM does have limitations in terms of material removal rates and surface finish. The process is generally slower compared to traditional machining methods, and a post-EDM finishing step might be required to achieve the desired surface quality. Despite these limitations, EDM remains a valuable technique for titanium alloys due to its ability to work with hard and intricate materials, where conventional methods often fall short.

*Abrasive Water Jet Machining* (*AWJM*) [150,151]: AWJM is a material removal process that is particularly advantageous for shaping and cutting titanium alloys and other hard-to-machine materials. Titanium alloys, with their high strength and resistance to heat, often benefit from the capabilities of AWJM. In this process, a high-velocity stream of water mixed with abrasive particles is directed towards the workpiece. The abrasive particles, often garnet or aluminum oxide, effectively erode the material due to their high kinetic energy. The controlled mixing of water and abrasive allows for precise material removal, and the process can accommodate a wide range of thicknesses and complexities. For titanium alloys, AWJM offers several benefits. Its non-thermal nature prevents the risk of heat-induced distortion or damage, which can be a concern when using traditional machining methods. Additionally, the absence of mechanical stresses associated with conventional cutting minimizes the risk of work hardening, which is common in titanium. AWJM is suitable for intricate shapes, stacked material layers, and composite structures present in titanium components used in aerospace, automotive, and medical applications. However, achieving a smooth surface finish might require secondary finishing processes, as AWJM can produce a slightly rough surface due to the abrasive action.

*Laser Machining* (*LM*) [152,153]: LM is a manufacturing process that holds significant promise for shaping and engraving challenging materials. For titanium alloys, LM has garnered attention due to its ability to overcome the difficulties associated with their high strength and heat resistance. In LM, a high-energy laser beam is focused onto the workpiece, where it melts, vaporizes, or ablates the material. The precision and intensity of the laser can be finely controlled to achieve intricate cuts, fine details, and complex shapes. Titanium alloys, with their ability to absorb laser energy effectively, make them suitable candidates for this technique. LM offers multiple benefits. Its non-contact nature eliminates tool wear, reduces mechanical stresses, and minimizes the risk of introducing impurities into the material. Additionally, the localized heat input minimizes the heat-affected zone, reducing the potential for distortion or residual stress. LM is well suited for aerospace, medical, and automotive applications where precision and minimal material loss are critical. However, achieving optimal results might require adjustments to laser parameters based on the alloy’s composition and thickness. Despite the clear advantages, LM may generate an undesirable surface roughness, necessitating post-processing steps to achieve the desired finish.

*Ultrasonic Machining* (*UM*) [154,155]: UM is a material removal method that offers unique advantages for shaping and machining hard materials. In the case of titanium alloys, UM addresses the challenges posed by their high hardness and toughness. UM involves the use of ultrasonic vibrations, typically in the range of 20 to 40 kHz, to facilitate the removal of material from the workpiece. A tool, often made of softer material than the workpiece, is pressed against the workpiece while ultrasonic vibrations are applied. The abrasive slurry consisting of abrasive particles suspended in a liquid helps in the material removal process. The non-thermal nature of the process prevents the introduction of heat-induced distortion or changes in the material’s properties. Overall, UM is suitable for applications where traditional machining techniques might struggle, such as aerospace components and medical implants. However, it might require longer machining times compared to conventional methods, and, as seen for other advanced machining techniques before, post-processing might be necessary to achieve the desired surface finish.

Machining titanium alloys requires careful consideration of cutting parameters, tool materials, tool geometries, and cooling/lubrication strategies to manage heat generation and tool wear [156]. The reactivity of titanium with cutting tool materials can lead to chemical reactions and wear, necessitating the use of suitable tool coatings. Additionally, proper chip control and effective removal of heat from the cutting zone are critical to ensure dimensional accuracy and surface quality. Moreover, the heat generated at the tool-workpiece interface can cause localized temperatures to rise. In the presence of oxygen, this elevated temperature can lead to the diffusion of oxygen into the titanium, causing, as discussed before, the formation of a thin layer of oxygen-rich alpha-phase titanium on the surface, often referred to as “α-case”.

## 7. Powder-Based Processes

In terms of powder-based processes, titanium alloys can be produced by both powder metallurgy techniques [157] and, more recently, additive manufacturing techniques [158]. In both cases, the powders can be produced as pre-alloyed [159] or by a blending process [160].

The powder metallurgy processes [159,161,162,163] usually require a shaping process followed by thermal cycles to extract any organic binders (de-binding treatment) and to consolidate the material (sintering). The last treatment is usually carried out at temperatures around 1250 °C, in an inert or vacuum atmosphere. The sintered material usually has good mechanical properties, also due to the controlled microstructure (the microstructure also depends on the powder quality), but often has some residual porosity. In this case, a post-treatment process is required to better consolidate the material using Hot Isostatic Pressing (HIP) [164,165]. The sintered component is usually impregnated at high temperature and pressure using inert gases to mechanically close the residual porosity.

Most of the emerging techniques for producing near-net-shape components using Ti powders are additive manufacturing (3D printing) processes.

The most commonly used techniques are electron beam melting (EBM) [166] and selective laser melting (SLM) [167]. In both cases, the part is created by selectively melting thin layers of powder using a heat source, which can be a laser or an electron beam. In the case of electron beam melting, the process is usually carried out in a vacuum, whereas in the case of SLM, the 3D printing is carried out in a protective atmosphere. The microstructure of 3D-printed parts is strongly influenced by the printing parameters and is completely different for the same material printed by the above-mentioned techniques. While additive manufacturing revolutionizes customization, it may lead to unintended microstructures due to rapid solidification, impacting mechanical properties and biocompatibility [143,144,145]. Stringent control and post-processing, including heat treatments such as hot-isostatic pressing and/or surface modifications, are often necessary in order to mitigate these concerns [146,147]. The as-printed 3D-printed part also has a different surface finish, which is better for the parts produced by SLM. A weak point in terms of mechanical properties is the presence of residual stresses [168,169], which have a strong influence on the fatigue properties of the components, especially for materials produced by the SLM technique. The main advantage of the use of additive manufacturing techniques is the production of components with complex geometries or composed of reticular structures. In particular, the use of reticular structures also makes it possible to control the stiffness of the component to match bone properties [158].

## 8. Biological Corrosion

One of titanium’s outstanding features is its biocompatibility, which refers to its ability to interact harmoniously with living tissues without eliciting harmful responses. This biocompatibility stems from the oxide layer that spontaneously forms on the surface of titanium upon exposure to air [170]. This oxide layer, primarily composed of titanium dioxide (TiO_2_), is stable, inert, and effectively isolates the underlying metal from the surrounding biological environment [171]. This phenomenon prevents corrosion and minimizes adverse reactions. In the context of bone and tissue integration, in particular, titanium exhibits excellent osseointegration—direct bonding between bone and implant surface [12]. The oxide layer’s surface chemistry fosters the adsorption of biomolecules like proteins [172], enabling enhanced cell attachment, proliferation, and differentiation. Cells, such as osteoblasts, adhere to the implant surface more readily, promoting bone growth and implant stability. A more in-depth analysis of titanium’s bioactive role will be presented in Section 10.

Alloying elements play a significant role in modulating the susceptibility of titanium alloys to biocorrosion in physiological environments [173,174,175,176]. While most titanium alloys are considered highly resistant to corrosion, certain factors, such as tribocorrosion [177,178] and fretting corrosion [179,180], can introduce complexities. Titanium’s surface, while inherently resistant to corrosion, is susceptible to wear, which can exacerbate corrosion effects in specific conditions.

Alpha-phase titanium alloys, characterized by a high proportion of alpha-phase crystals, are generally more resistant to corrosion due to their stable microstructure [181,182]. In contrast, beta-phase titanium alloys, rich in beta-phase crystals, can be more prone to corrosion, especially in aggressive environments.

**Figure 4:**
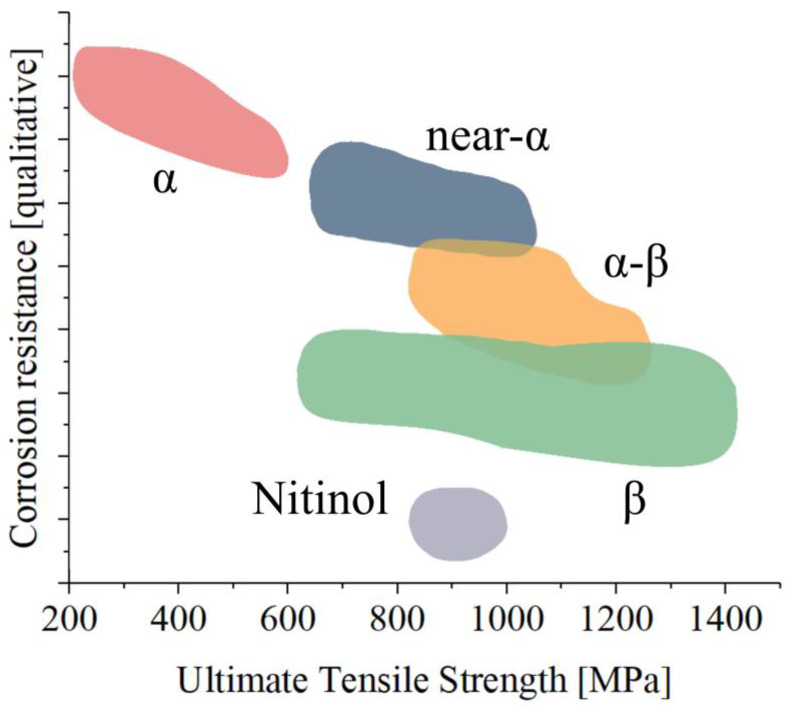
Relationship between the ultimate tensile strength and the qualitative corrosion resistance of titanium alloys applied in the biomedical field. The colored areas indicate the kind of alloys.

Concerns have arisen regarding the potential release of trace elements like vanadium or aluminum from titanium alloys, which could have adverse effects. However, current reports suggest that these releases are minimal, particularly when wear is not a factor. Figure 4 summarizes the relationship between ultimate strength and qualitative corrosion resistance for the most common titanium alloys applied in the biomedical field [43,183].

To enhance the corrosion resistance of titanium alloys, alloying elements such as molybdenum and ruthenium can also be introduced. These elements have demonstrated their ability to improve the alloys’ long-term stability in biological environments. However, the interaction between corrosion resistance, alloy composition, and wear dynamics underscores the intricacies of achieving optimal performance in various medical applications [184,185].

In general, Ti alloys are the biometals less prone to ion release in the human body due to the high stability of the passive films combined with the fastest regeneration of the passive film if destroyed by mechanical damage. No Ti-containing biomolecules have been found in tissues or body fluids in contact with Ti alloy implants [186].

## 9. Biomedical Applications

Depending on function, size, shape, and anatomical location, different titanium alloys are applied for different types of biomedical devices. The types of biomedical applications will be discussed in this section, and their list can be found in Table 3.

### 9.1. Dental Implants

The integration of titanium into dental implants and orthodontic braces has revolutionized both fields, significantly improving patient outcomes and comfort [187]. The application of titanium in dentistry finds its roots in the pioneering work of Dr. Per-Ingvar Brånemark, who discovered osseointegration—the direct bond between bone and a titanium surface. This breakthrough not only transformed the landscape of dental implants but also set the stage for the use of titanium in orthodontic applications, such as braces [188].

Titanium dental implants have emerged as the gold standard for replacing missing teeth, in particular as posts [189,190]. The application of titanium posts involves the surgical placement into the jawbone, where, over time, they integrate with the surrounding bone tissue, progressively increasing stability. This integration provides a sturdy foundation for prosthetic teeth, restoring mechanical strength and stability [191], while the upper part of the implant, called the “crown” and made of ceramic or composite materials, restores aesthetics and functionality. Dental implants not only improve chewing and speech but also prevent bone loss, preserving facial structure and overall oral health.

Titanium alloys are also pivotal in orthodontics, where they are used to create braces, wires, and other orthodontic appliances [192,193]. Titanium’s exceptional strength-to-weight ratio and corrosion resistance make it an ideal choice for devices that apply controlled forces to move teeth into proper alignment. Titanium’s biocompatibility ensures that patients tolerate orthodontic treatment well, leading to effective and predictable outcomes.

The oral environment presents a dynamic and challenging milieu for dental implants and orthodontic devices. The oral cavity experiences significant pH fluctuations due to the consumption of various foods and beverages [194]. Acids from acidic foods and drinks can erode tooth enamel and potentially affect the surface of dental devices [19]. Titanium’s corrosion resistance is paramount in combating this challenge, preventing the degradation of the implant surface and ensuring long-term stability.

The oral environment harbors diverse microbial populations, including bacteria. Bacterial colonization on implant surfaces can lead to the formation of biofilms, which pose a threat to device longevity [195,196,197]. While titanium’s biocompatibility discourages bacterial attachment, coatings or modifications may be applied to further deter biofilm formation, ensuring the health of surrounding tissues and the longevity of implants.

Moreover, while mechanical solicitations in the oral environment are generally less severe and geometrically complex compared to orthopedic applications, they are not to be underestimated. Forces from chewing, biting, and speaking subject dental devices to cyclic loading [198,199,200]. Materials must possess sufficient mechanical strength and fatigue resistance to withstand these repetitive stresses over the long term without compromising device integrity.

Long-term clinical studies underscore the success of titanium-based dental devices. Dental implants exhibit impressive survival rates, with 10-year success rates exceeding 95%, according to systematic reviews [201,202,203,204,205,206,207]. Orthodontic appliances made from titanium alloys demonstrate consistent performance, enabling orthodontists to achieve desired tooth movements with a high degree of precision.

Three are the most common alloys applied in the dental field:

*β-titanium alloys* [192,208,209,210]: These alloys, primarily investigated for dental wires due to their mechanical properties, notably elastic modulus and deformability, encounter limited commercial application;

*Ti-6Al-4V* [211,212,213,214]: For applications that require very high mechanical strength without compromising biocompatibility, such as in bone plates, screws, fasteners, abutments used for oral implants, and brackets for orthodontic appliances;

*Commercially pure titanium* [215,216,217,218]: Commonly used for orthodontic wires due to its flexibility and gentle force delivery; it is also often applied in orthodontic brackets, archwires, abutments, frameworks for partial denture, and temporary crowns or bridges;

*Nitinol* [219,220,221]: Nitinol’s remarkable shape memory and superelastic properties make it particularly well suited for orthodontic archwires, but it is also applied for orthodontic springs and expansion devices.

### 9.2. Joint Replacement

From joint replacements to fixation components, titanium’s properties address the complex demands of the musculoskeletal system. This material has enabled the development of implants that seamlessly integrate with bone [13,222], facilitating improved mobility, function, and quality of life for countless individuals. Titanium’s impressive strength, in particular, allows for the creation of robust implants that can withstand physiological loads, ensuring stability and longevity in orthopedic applications [223]. In the history of orthopedic materials, similar results were previously achieved using cobalt–chromium alloys, but titanium presents two critical advantages: it can osseointegrate more easily and it has a higher strength-to-weight ratio, meaning that titanium implants are lighter than cobalt–chromium ones [224,225].

Titanium is used in various joint implants, such as shoulder [226], elbow [227], wrist [228], hip [229], knee [230], and ankle [231]. However, a distinctive aspect of titanium’s use in these implants is that it is never employed as an “articulating component.” This means that titanium is not utilized for the surfaces that directly come into contact and rub against each other within a joint during movement.

The reason for this selective application lies in titanium’s inherent characteristics. While titanium exhibits exceptional biocompatibility, strength, and corrosion resistance, its performance in terms of wear [232] and tribo-corrosion [178] is limited. Titanium’s surfaces are more prone to wear and degradation when subjected to the repetitive, sliding, and abrasive forces present in articulating joint components. This could potentially lead to increased friction, wear debris generation, and even corrosion, which are undesirable outcomes for joint implants aiming to mimic the natural joint’s smooth and efficient movement.

Instead, titanium finds its niche in orthopedics as a material choice for “load-bearing components” within joint implants. For instance, in hip replacements, titanium is often used for the creation of the femoral stem [233] and the acetabular cup [234]. These load-bearing components provide stability and support, transferring the mechanical load from the implant to the surrounding bone. The unique combination of titanium’s flexibility, strength, and light weight is particularly advantageous in these applications, as it enables the implant to withstand physiological loads without compromising the structural integrity of the implant–bone interface.

Apart from joint prostheses, titanium is also used for other, not articulating bone implants, such as ribcages [235] and skull [236] implants, spinal cages, bone scaffolds [237], and support devices. Unlike articulating joint components, which undergo repetitive motion and friction, non-articulating bone implants pose minimal risk of tribological damage. As there are no moving parts or surfaces in contact, the potential for wear, friction-induced debris, or corrosion is significantly reduced. This enhances the longevity and reliability of these fixed devices.

There are various titanium alloys that have been approved for orthopedic uses, but only a limited number of them are utilized in the devices that are actually commercially available. The list is as follows:

*Ti-6Al-4V* [223,238,239], which is the most widely used titanium alloy in orthopedics, is applied in various load-bearing applications, including hip and knee implants. One of the drawbacks of using Ti-6Al-4V is the potential ion release, as discussed in Section 6, but other concerns include the relatively high elastic modulus, which can be responsible for stress shielding phenomena and consequently bone resorption.

*Ti-6Al-7Nb* [240,241,242] has a similar biocompatibility and a lower elastic modulus when compared to Ti-6Al-4V, but also a lower mechanical strength. Additionally, its microstructure is more difficult to control.

*Ti-15Mo* [92,243,244] is used in orthopedics for its corrosion resistance and lower modulus of elasticity. It is employed in applications like spinal implants and bone screws. Its strength is lower compared to Ti-6Al-4V, which may limit its use in high-load scenarios. There are also concerns about the potential release of molybdenum ions in the surrounding biological tissue.

*Ti-13Nb-13Zr* [245,246,247] is utilized for its biocompatibility and lower Young’s modulus. It is employed in spinal implants, bone plates, and dental applications. It has relatively lower strength compared to other titanium alloys, which can limit its use in high-stress applications.

*Commercially pure titanium* [248,249,250]: CP Ti is employed for non-load-bearing applications like fixation screws, wires, and plates. Its lower modulus of elasticity reduces stress-shielding effects. Despite the superior biocompatibility, it lacks the high strength of titanium alloys, restricting its use to applications with lower mechanical demands.

*Nitinol* [251]: the intermetallic compound Nitinol is applied in Mitek suture anchors, which are used to attach soft tissue to the bone.

Overall, titanium alloys used in orthopedic applications have shown very successful clinical outcomes, with some joint implants surviving as long as 30 years or more, despite the challenging environment.

### 9.3. Trauma Devices

Trauma devices, such as bone plates, screws, and intramedullary nails, require materials that can withstand the mechanical stresses imparted on them within the human body, which can be superior to those faced by orthopedic devices. Moreover, when compared to orthopedic implants, trauma devices are applied in a broader range of anatomical locations and the specific amount of mechanical stress they will encounter can be hard to predict. For this reason, only a limited selection of materials is usually applied in trauma devices, in particular, cobalt–chromium, stainless steel, and titanium, with the latter being the least mechanically performing, in particular, under cyclic fatigue conditions: in a 1996 review of implant failures, the authors noticed that trauma devices made of titanium had five times higher failure rates due to fracture when compared to similar implants made of stainless steel [252] and recommended that the use of titanium devices be limited to less challenging applications. Perren et al., in 2017, noted that titanium plates have generally superior biological properties, have lower chances of adverse reactions, and carry lower risks of infection [253,254]. It must be noted that mechanical irritation through movement of the tissues in relation to the implant surface is also a potential cause for adverse reaction, but it is independent from the material used.

The design of trauma devices [255] with respect to the anatomical location is a key aspect for the success of titanium implants, in particular considering that titanium is a soft metal that can easily produce cytotoxic particulates due to abrasive wear [256].

In screws, including those applied in trauma, the mechanically weakest location is the root of the thread, but thread fractures have never actually been reported in the biomedical literature [257]. Screw shaft fractures, on the other hand, are relatively common. This indicates that the contact forces between bone and thread are relatively weak when compared to the bending solicitations that the shaft faces.

It must be noted that the stress distribution varies greatly in both screws and plates when comparing locking and conventional plates. In locking plates, screws are anchored to the plate itself. Unlike traditional plates where screws rely on friction to hold the bone fragments together, locking plates have special threaded screw holes that completely lock the components together. This locking mechanism provides more stability and minimizes the risk of screw loosening or backing out over time, but it also increases the amount of mechanical stress that both bone and device have to bear [258]. Locking plates are particularly useful in situations where bone quality is poor, as they can provide better fixation in osteoporotic or compromised bone, but they can also cause severe complications such as damage to the surrounding tissues [259].

Alloys applied in trauma devices are limited to Ti-6Al-4V [69,260], Ti-6Al-7Nb [260], and commercially pure titanium [261,262].

### 9.4. Spinal Implants

As previously stated (Section 1), spinal implants are somewhat in the between arthroprosthetic and trauma devices, and their required properties can vary greatly depending on the specific application. Titanium pedicle screws are used to stabilize and immobilize the spine: they are inserted into the vertebral pedicles and serve as anchors for other spinal implant components. Among all the spinal implants, pedicle screws are the ones that have the worst survival rate in vivo [263,264,265], as the diameter of their shaft is limited by the anatomy of the vertebrae, resulting in localized stress intensification.

Titanium rods are often used to connect pedicle screws and provide additional stability to the spine, but due to the higher resisting section, these components are less likely to fail due to mechanical stress. These rods can be contoured to match the curvature of the spine, and this process can lead to fatigue cracking [266], but rod fractures are so uncommon that no systematical follow-up data are available in the literature.

Titanium interbody cages [267,268,269] are used in anterior lumbar interbody fusion (ALIF) and other spinal fusion procedures. They are placed between adjacent vertebrae to restore disc height and promote fusion.

Titanium alloys are also used in the construction of artificial discs, also known as disc replacements or disc prostheses [270,271,272]. These devices are designed to replace damaged or degenerated intervertebral discs and restore spinal motion.

### 9.5. Cardiovascular Devices

Titanium and its alloys play a crucial role in the development of cardiovascular devices, contributing to improved patient outcomes in the treatment of various heart and vascular conditions. These alloys possess properties that make them well suited for devices aiming to restore normal blood flow, enhance cardiac function, and provide structural support.

Several cardiovascular devices incorporate titanium alloys, including coronary and peripheral vascular stents, devices that are designed to open narrowed or blocked arteries, restoring blood flow and preventing complications like heart attacks, as well as artificial mechanical heart valves, which replace damaged or dysfunctional native tissue and ensure proper blood flow through the heart chambers.

Titanium’s biocompatibility and corrosion resistance make it an excellent choice for enclosures that house pacemakers and implantable cardioverter-defibrillators (ICDs). These enclosures protect sensitive electronics from both the chemically aggressive biological environment and external electromagnetic interferences [273], while minimizing the risk of immune responses [274]. Unlike materials applied for arthroprostetic and trauma devices, the alloys used in pacemakers and defibrillators do not require high mechanical resistance, and the choice of using titanium over other materials is driven purely by chemical resistance and insulation capabilities. Titanium can also be utilized for the electrode tips [275], as an alternative for platinum iridium, titanium nitride, and, more recently, nano-porous carbon [276].

The high mechanical properties of titanium alloys are crucial for other cardiovascular applications, such as in the case of stents and mechanical heart valves.

Cardiovascular stents are medical devices designed to treat various cardiovascular conditions, primarily involving the arteries and blood vessels. These small, tube-like structures are used to help maintain the patency (openness) of narrowed or blocked blood vessels, ensuring a consistent and unobstructed blood flow.

Stents are basically expandable tubes of mesh that are plastically deformed into position. Apart from titanium, good clinical results have been achieved by using AISI 316 Stainless Steel, platinum–iridium alloys, tantalum, and cobalt–chromium alloys [277]. Metals are the main materials utilized for stents because of their mechanical properties and visibility on X-ray imaging [278], but in order to prevent stent restenosis—the re-narrowing or recurrence of blockage in a previously treated blood vessel—the metallic scaffolds are usually coated with hard and anti-adhesive layers such as titanium oxide, titanium nitride [279], and titanium oxynitride [280].

Heart valves present several similitudes with stents, as they are also subjected to mechanical load and require additional coatings to prevent biological interactions. Unlike stents, heart valves are mainly subject to cyclic fatigue and wear [281]. In order to prevent cellular adhesion and proliferation on the valve surface, which might potentially result in an obstruction, heart valves are usually coated with an anti-adherent hard coating such as nanocrystalline diamond [282], diamond-like carbon [283], or titanium oxide [284].

Three titanium alloys have found application in cardiovascular devices:

- *Ti-6Al-4V* [285,286,287], which possesses the best combination of mechanical strength and corrosion resistance;
- *Commercially pure titanium* [288,289,290] (Grade 2 in particular), which has higher corrosion resistance, biocompatibility, and can be easily plastically deformed;
- *Nitinol* [291,292,293], which possesses both shape memory and super elasticity and can be easily compressed for delivery through a catheter and then self-expand once it reaches the desired location.

### 9.6. Soft Tissue Implants

Titanium is not commonly used as the primary material for soft-tissue implants, which are typically made from materials that are more compatible with the characteristics and flexibility of soft tissues, such as collagen, silk, and various polymers. Soft-tissue implants are designed to mimic the properties of natural soft tissues, such as skin, fat, and muscle, and they are often used for reconstructive or cosmetic surgery.

Despite being more commonly associated with hard-tissue implants, such as those used in orthopedic surgery and dental implants, titanium may, in some cases, be used in combination with other materials or as a component of soft-tissue implants for structural support or anchoring purposes. For instance, titanium meshes may be used to provide support for soft tissues during the healing process or repair [294,295,296]. In soft tissue applications, titanium is commonly utilized as a mesh of wires or, less commonly, as a porous scaffold [297].

Both meshes and scaffolds have superior flexibility when compared to bulk materials, and can display elastic moduli orders of magnitude lower, even if not as low as the soft tissues they are in contact with. As a further drawback, porous structures display lower ultimate strength than their bulk counterparts, which might result in premature failures, in particular, under bending conditions.

## 10. Biological Functions

The low solubility of titanium in water has been considered the main indicator of its biological inertness. In an oxidizing atmosphere, titanium exists as Ti(IV), and in an aqueous solution is extremely prone to hydrolysis. At the pH of blood (pH 7.4), Ti(IV) compounds dissociate and transform into titanium dioxide, which is considered to be basically insoluble (0.2 fM at pH 7.4) [298,299]. As for other metals, binding by biomolecules in biological fluids actually increases solubility, and several studies suggested values up to about 1 μM in whole blood [300,301]. Some authors have postulated that Ti(IV) can bind to the Fe(III) transport protein sTf, leading to its high organelle bioaccumulation without inhibiting Fe-dependent processes and causing toxicity, while careful delivery can exhibit functions comparable to those of iron [300].

Despite the potential cytotoxicity, since the time of Brånemark, hundreds of thousands of titanium implants have been osseointegrated without complications. This can be explained by taking into consideration that even if soluble, the amount of Ti(IV) released from TiO_2_ surfaces is virtually negligible, from a biological point of view. It has been suggested that titanium oxide’s capacity for osseointegration is a consequence of the high dielectric constant, which does not cause protein denaturation [302]. Early investigations also supported the hypothesis that the oxide layer incorporates both organic and inorganic material during its growth in vivo [303], providing a transition region between the “pure” implant and the organic material [304]. Molecular dynamics simulations showed that both hydroxylated and nonhydroxylated TiO_2_ surfaces result in protein–surface electrostatic interactions that are responsible for absorption [305], and the process is faster for hydroxylated surfaces. Experimentally, various proteins such as albumin [306], fibronectin [307], and laminin [308] have been found able to absorb onto titanium dioxide surfaces, proving that adhesion and subsequent osteointegration are indeed regulated by the nanometric passive layer on the outermost surface of titanium implants. Recent publications suggest that the interaction between TiO_2_ surfaces and proteins is controlled by (I) electrostatic interactions, (II) hydrogen bonding, (III) hydrophobic interactions, and (IV) van der Waals forces:

Divalent cations (mainly calcium) binding can act as a bridge between the negatively charged protein sites (for example R-COO^−^) and the negatively charged TiO_2_ surface [309,310];

It was observed that TiO_2_ bonds with positively charged R-group (lysine, arginine, and histidine) and nonpolar aliphatic R-groups amino acid, but has less affinity for aromatic R-group, polar uncharged R-group, and negatively charged R-group containing amino acids, which suggests that the affinity of TiO_2_ for amino acids depends on the ability to form stable hydrogen bonds [311,312];

Controlling the hydrophobicity of TiO_2_ surfaces can increase or reduce protein adhesion and modified hydrophilic TiO_2_ surfaces displayed anti-fouling capabilities [313];

In most scenarios, Van der Waals forces are complementary to hydrogen bonding, so it is not surprising that both phenomena are usually coupled together [314,315,316].

Apart from cellular adhesion, titanium and, in particular, TiO_2_ were also reported to be able to stimulate cellular proliferation, probably due to the production of reactive oxygen species (ROS) [315], but, on the other hand, ROS generation induced by TiO_2_ particles might directly or indirectly damage DNA to cause genotoxicity and impact cellular signaling pathways to modulate cell proliferation, resulting in irreversible cell transformation [317].

Titanium oxide nanoparticles present a high surface area per unit of mass, which in turn increases ROS production and results in a much higher genotoxicity risk [318,319] when compared to bulk titanium implants.

Moreover, as with most biomedical materials, titanium implants elicit an initial inflammatory response as part of the wound-healing process. The interactions include platelet adhesion and mononuclear cell attachment, while mononuclear cells or macrophages interact with the implant and release inflammatory cytokines and chemokines [320]. While an inflammatory response is unavoidable, the levels of cytokine are dependent on many factors, such as anatomical location, surface morphology, chemical composition, and patient conditions [321,322,323].

It has also been observed that the initial adhesion of primary osteoblasts to titanium involves the activation of similar intracellular signaling pathways and gene expression as fibronectin [324] and surface morphology can be further modified to activate phospholipase D1 [325] and other enzymes, meaning that titanium (or better, TiO_2_) also plays a role in controlling signaling pathways.

## 11. Long-Term Survival Rates

### 11.1. Causes of Failure

**Figure 5:**
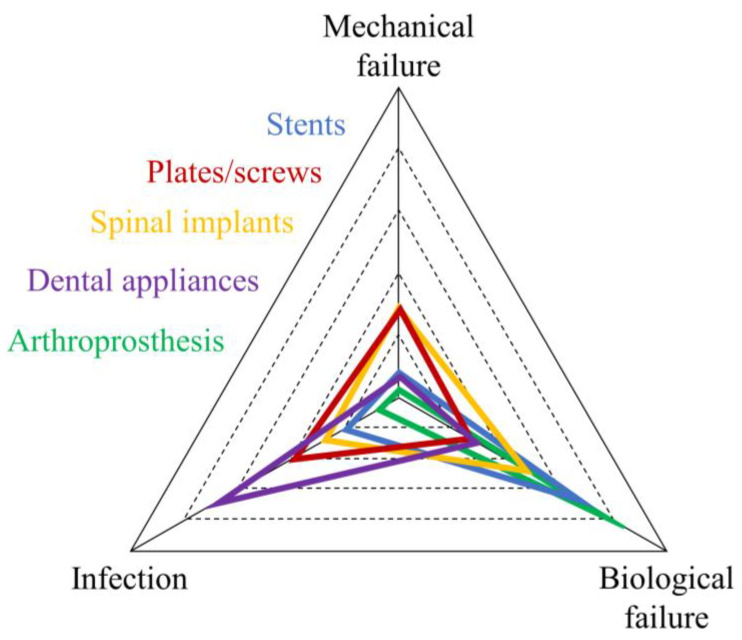
Implant failures divided by category, going from 0% (in the center of the triangle) to 100% (at the outermost corners) of the failures.

Titanium implants have a history of long-term success, with devices being able to last up to half a century without the need for revision. Nevertheless, the success of titanium implants strongly depends on how the performances are evaluated and, overall, varies greatly between different applications. For example, the successfulness of a titanium stent depends on the extent of restenosis and the time that occurred between implantation and the next adverse episode, while the successfulness of a spinal device is often evaluated considering how much influence it had on the posture of the patient. Figure 5 resumes the causes for implant failure by splitting them into three categories: mechanical failures such as implant deformation or fracture [326,327], biological failures [328], such as lack of integration or presence of extensive restenosis [329], and infection-related.

All three can occur on all types of biomedical devices, but not with the same probability. Moreover, survival rates greatly depend on the health status of the patient, where age also plays a key role.

### 11.2. Clinical Follow-Ups

**Figure 6:**
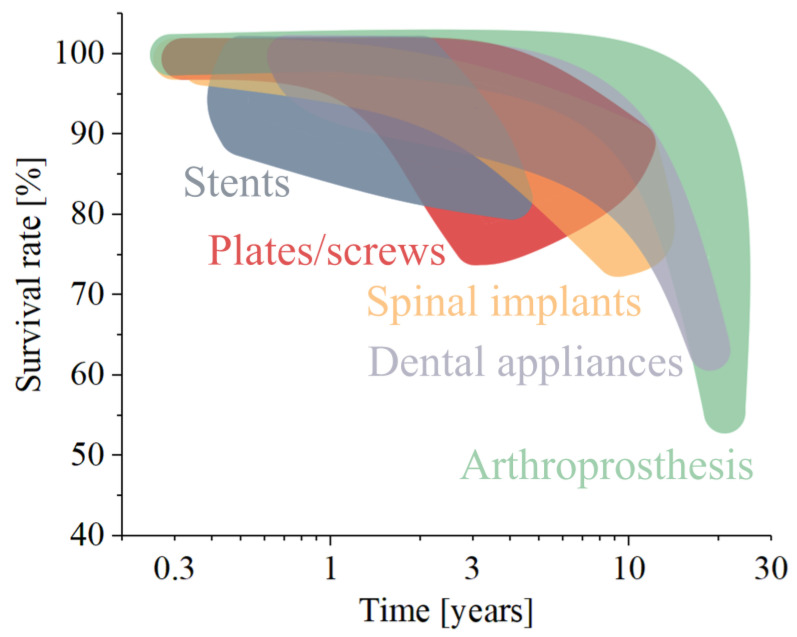
Survival rate over time for different categories of titanium biomedical devices.

The results of all clinical follow-ups mentioned in this review for patients who were treated with titanium-based biomedical devices are summarized in Figure 6. The type of device has been divided into five categories based on similarity, to simplify interpretation. The results are not divided by cause of revision, which will instead be discussed case by case in the text.

It can be observed that most devices, with the noticeable exception of stents, have a survival rate close to 100% at least in the first few months after implantation. In most cases, early revisions are caused by infections and infections are, overall, relatively uncommon in modern scenarios. Orthopedic implants, for example, reported infection rates below 1% in the first 2 years, in particular for hip articulation [330,331]. Still, risk factors such as obesity, age, presence of diabetes, smoking habits, additional nosocomial infections, wound contamination, preoperative stay, and number of operations were associated with increased risks [332,333,334,335]. The influence of these risk factors has predominantly been explored in the field of orthopedic surgery, where the larger patient cohort provides increased statistical robustness for correlation analyses. Nevertheless, it is reasonable to anticipate that analogous outcomes would likely prevail for various other implant types.

Two additional factors that are often associated with implant infection rates are the geometry of the device (often referred to as “complexity”) [336,337] and the depth of the incision, as implants that are applied closer to the skin seem to incur increased risks of infection [338,339], but statistical correlations have yet to be demonstrated.

For stents, the first few months after implantation appear to be the most critical, as survival rates as low as 87% were reported within the first year [340,341,342,343,344,345]. Most of these early failures appear to be caused by misplacement and migration or in-stent restenosis, but mechanical failures (fractures or inadequate expansions) and infections were also frequently reported.

The region of Figure 6 between a few months and the first 3 years shows more marked differences between the different categories of implants, and the largest statistical dispersions within the same group. Plates and screws, for example, see survival rates as low as 75% and as high as 95% at about 3 years’ follow-up. The statistical dispersion between reported survival rates is mainly associated with patient conditions: specifically designed plates are often used during the reconstruction of bone tissue affected by tumors [346,347], which occasionally results in flap necrosis, plate extrusion, or even fractures [347,348]. Similarly, nails and screws [263,264,265,349,350,351] used in the treatment of metastatic bone fractures can encounter premature failures due to the weakened condition of the surrounding bone tissue [352]. Apart from biological and mechanical failures, the treatment of bone tumors with metallic devices can also result in higher risks of surgical site infections [353].

Low survival rates at about 3 years after implantation were also occasionally reported for stents, and this is again associated with the specific patient cohorts [340,341,342,343,344,345]. Patients requiring a stent often exhibit multiple risk factors that increase the likelihood of implant failure, including conditions such as obesity, diabetes, hypertension, smoking, and a history of cardiovascular diseases [354,355].

In the last region of the graph, going from about 5 to 30 years’ follow-up, most published reports are focused on spinal, dental, and arthroprosthetic implants. These three categories are considered to be the most durable and reliable, but within 10 years, survival rates as low as 70% have been reported for spinal appliances, in particular when associated with vertebral resection due to the presence of tumors [356]. It should also be taken into account that spinal implants have a broad range of scopes, and, consequently, the conditions and probabilities for failure and revision vary greatly. In the treatment of spinal deformities, for example, the outcomes are conventionally assessed by radiographic parameters such as curve size, thoracic and lumbar sagittal plane, and coronal and sagittal balance, which have little to do with the performance of the materials utilized [357]. Artificial discs [358], on the other hand, are probably the best example of spinal devices that have high survival rates at long-term follow-ups. Still, even for reliable implants, the long-term survival rates of spinal implants are in the range of 90% at 10 years [359,360,361].

Despite facing similar potential complications [362,363], dental implants have higher survival rates at longer follow-ups when compared to spinal implants. This is probably caused by the differences in mechanical loading, implant mobility, and also in the conventional failure criteria. Titanium dental implants are often subject to fatigue loading and fretting [364,365], but the stress fields involved are simpler (mostly compression, in the most simple case of titanium posts) and the forces involved are lower [366,367]. Moreover, in the presence of weakened bone tissues, it is relatively easy for the patient to change habits and diet in order to delay the failure of the implant [368,369]. From the chemical and biological point of view, the oral environment is considered to be more aggressive [370,371] than the inside of the body, but also more forgiving.

The longest follow-ups are only available for arthroplasty devices, and most of the reviews published in the literature are actually focused on the hip joint. Results clearly show that titanium arthroprostheses have a high survival rate up to about 10 years of follow-up, but then show a steep decrease between 10 and 30 years. These devices are extremely reliable, and the failure is prevalently caused by patient-associated conditions, such as advanced age [372,373,374,375,376,377,378,379,380,381,382,383,384,385,386,387,388]. Most late failures are caused by osteoporosis and other risk factors that were not present (or not as advanced) at the time of implantation and, overall, mechanical failures and infections only account for about 15% of the total [389].

## 12. Surface Modification of Titanium

Surface modifications play a crucial role in enhancing the performance and biocompatibility of titanium and its alloys in biomedical applications. In this section, we will provide a list of common strategies for the improvement of the biocompatibility of titanium alloys.

*Passivation/Oxidation/anodization:* The common aim of these three families of treatments is to control the thickness and chemical composition of the oxide layer that is spontaneously formed on the outermost surface of all titanium alloys. For thin oxide layers, the most important characteristics are chemical stability and uniformity, as their main function is to protect the metal underneath, but when the thickness of the oxide increases to hundreds of nanometers or even microns, the layer can provide additional protection against abrasion and wear, and can be also functionalized with bioactive elements such as calcium and phosphorous [390]. Moreover, as the titanium oxide layer is responsible for protein adhesion and absorption, controlling the thickness and chemical composition can speed up wound healing and implant integration. With the anodizing process, it is also possible to produce nanotubes with controlled dimensions that can increase the surface properties of the implant by enhancing the cell-bone adhesion. In addition, the nanotubes can also be filled with antibacterial medicines to prevent infections.

*Hard coatings:* Various hard coatings have been applied to titanium alloys, in particular using CVD and PVD, and initially with the intent of increasing the poor tribological resistance [391], in order to be able to use titanium for articulating components such as femoral heads [392,393]. The gap in elastic modulus between the coating and the substrate resulted in early delamination [15] or chipping [394], and consequently an increase in abrasive wear. More reliable results could be achieved by combining together diffusive treatments and PVD coatings [395], but at the expense of fatigue resistance due to the increase in the titanium grain size. In modern applications, hard coatings are mainly applied to reduce cellular adhesion and prevent restenosis in cardiovascular devices [396,397].

*Bioactive coatings:* Apart from hard coatings, inorganic materials can also be deposited on titanium alloys in order to improve the biological interaction. These coatings can be deposited using a wide array of different techniques, going from plasma spray to electrodeposition, but the most commercial bioactive coatings can basically be referred to in three categories: titanium oxides, calcium compounds, and bioglasses. The properties of TiO_2_ coatings and their functionalization have been previously discussed in Section 10 and Section 12: Oxidation. For calcium compounds, they represent the most common and successful category of bioactive coatings applied on titanium alloys and include oxides, titanates [398], carbonates [399], phosphates [400], and apatites [401], all of which can be further functionalized by adding small amounts of other atoms or by regulating their crystallinity [402]. Calcium compounds are used in particular for bone integration and regeneration, as they share chemical similarities with the mineral fraction of bone tissues.

*Diffusive treatments:* Diffusive treatments, such as nitriding and carburizing [395], involve introducing nitrogen or carbon into the surface layer of the titanium alloy through a controlled diffusion process. This creates a hardened surface layer with improved wear resistance, reduced friction, and increased surface hardness. Due to the barrier effect caused both by the native TiO_2_ layer and the newly formed TiN (or TiC), the diffusion process can take hours or even days before completion [403]. The required time can be reduced by using additional energy sources such as lasers [404], plasmas [405], or ultra-high frequency induction heating [406], but even optimized processes have limitations due to the increased surface roughness [407] and can act as a crack triggering point in fatigue conditions [408].

*Physisorption and chemisorption*: Growth factors or other biomolecules can be immobilized on the surface of titanium to promote cell attachment, proliferation, and differentiation. This can be achieved through covalent bonding or physical adsorption, and can improve the biological response of the cellular solid in hundreds of different ways [409,410,411]. Such treatments are so different in nature, chemical composition, and application that they will not be discussed in detail in this review.

## 13. Challenges and Future Directions

**Figure 7:**
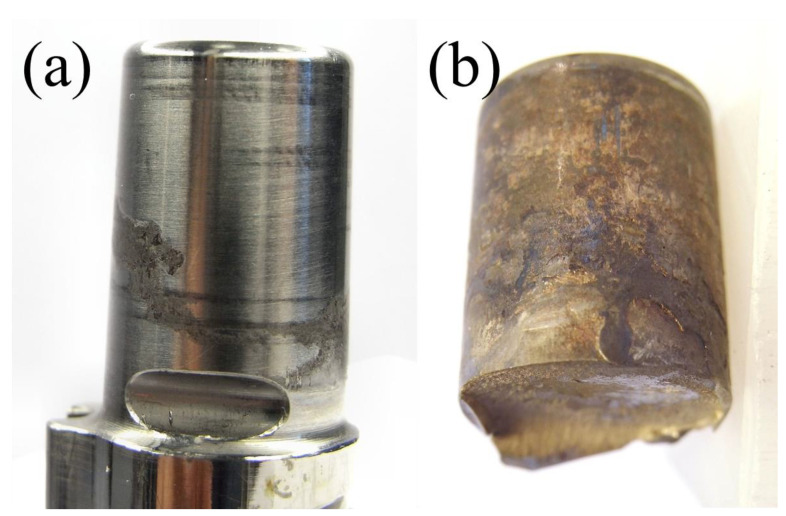
Examples of (**a**) fretting damage after 5 years in vivo and (**b**) mechanical failure due to fretting corrosion after just 2 years in vivo for Ti-6Al-4V tapers in modular femoral stems.

While titanium and its alloys have significantly contributed to the advancement of various biomedical fields in the last 60 years, several challenges still persist. First of all, titanium’s relative softness can lead to wear and fretting when in contact with other components (Figure 7). This is not only a major concern for load-bearing implants and articulating surfaces, but also for modular implants where titanium components are connected and interlocked with each other or with harder materials [412,413,414].

Fretting and wear could be reduced by using harder materials, but achieving sufficient hardness while maintaining biocompatibility has been historically challenging. Surface treatments like nitriding [415] and coatings aim to address this issue, but the former requires a long time and often compromises the microstructure, reducing the fatigue resistance and lowering the reliability of the implant in the long term, while the latter often delaminate due to the difference in elastic modulus, also causing third body wear in the process.

Achieving durable and uniform surface modifications that maintain their properties over time remains a challenge and even if new solutions are constantly proposed in the scientific literature, they seem to never reach the necessary maturity to be transferred to industrial applications.

While titanium’s inertness reduces infection risks, biofilm formation also remains a concern [196], in particular for modern, cellular solid materials and scaffolds that present a high area per unit of volume [416]. These devices can be hard to sterilize using “directional” sources like UV or gamma rays [417].

Despite being more complex to sterilize, cellular solids and scaffolds can mimic the structure and properties of natural tissues to encourage seamless integration and minimize foreign body response. This is in particular possible thanks to the various additive manufacturing technologies nowadays available on the market [418]. The constant improvement of such techniques will also create new potential applications for titanium alloys, in particular for custom-made devices, while the increase in achievable resolution will open new market opportunities for smaller implantable devices and sensors.

## 14. Advantages and Disadvantages

As we have seen in the previous sections, titanium and its alloys have become indispensable materials in biomedical applications, contributing significantly to the success of various medical devices and implants. In particular, independent of their application, titanium alloys exhibit excellent biocompatibility [419], minimizing the risk of adverse reactions when in contact with biological tissues, even under challenging biological loads and often decades after they were implanted. Moreover, unlike other successful structural biomaterials such as alumina, titanium alloys are not simply bio-inert, as they have been proven to be able to osseointegrate [420], providing additional support and stability to the implant over time.

Despite the extremely positive outcomes and the encouraging results of decades-long follow-ups, the use of titanium alloys in the biomedical field does have a few major disadvantages: titanium is expensive to produce [421] (about 15 times more expensive than medical-grade AISI 316L stainless steel), difficult to machine, easily damaged by wear, and also much heavier and stiffer than bone. Moreover, concerns are still raised about its biological stability, either because of the potential release of harmful ions over time or because of the low but not negligible risk of allergic reactions [422].

Over the years, innovative alloys with lower elastic modules [423], but not containing harmful elements, were developed. Ti-13Nb-13Zr [245,246,247], for example, has comparable mechanical properties but superior biocompatibility and a lower elastic modulus when compared to the most common Ti-6Al-4V alloy. Extensive research and experience have accumulated over decades with Ti-6Al-4V, leading to well-established manufacturing processes, quality control procedures, and design guidelines. In contrast, alloys like Ti-13Nb-13Zr are still relatively new, with ongoing research and development [424].

Other biocompatible metallic alloys, such as cobalt chromium and stainless steel, have superior wear resistance and mechanical properties, but also higher elastic moduli and, overall, generate more concerns about their biocompatibility [425]. In considering alternative materials for various biomedical applications, high-performance polymers are a viable option [426]. These polymers offer a compelling combination of good biocompatibility and mechanical properties that can closely mimic those of natural bone [427,428,429]. Moreover, the ease with which they can be functionalized enhances their versatility in biomedical engineering [430,431]. While high-performance polymers present promising alternatives for many practical applications, there are instances where the mechanical robustness of titanium makes it the more reliable choice, such as femoral stems or nails.

## 15. Conclusions

The evolution of titanium and its alloys in the biomedical field has transformed the landscape of medical engineering and patient care. From the pioneering work of Dr. Per-Ingvar Brånemark to the present day, titanium’s exceptional properties have catalyzed innovations in orthopedics, dental care, cardiovascular interventions, soft tissue implants, and beyond. The exceptional combination of the biocompatibility, corrosion resistance, and mechanical strength of titanium has enabled the development of implants that seamlessly integrate with the body, promoting healing, mobility, and a better quality of life for patients.

However, as this review has highlighted, challenges persist. The field is actively addressing concerns such as wear, surface modifications, infection prevention, and the need for improved integration with soft tissues. The quest for innovation remains fueled by a pursuit of durability, precision, personalization, and enhanced functionalities.

As researchers, clinicians, and engineers bridge disciplines, synergies emerge, bringing forth solutions that redefine the boundaries of medical possibilities. Titanium’s legacy in the biomedical field stands as a testament to human ingenuity, the pursuit of excellence, and the unwavering commitment to advancing healthcare for the betterment of humanity. As this remarkable journey unfolds, titanium remains a steadfast ally, propelling medical science toward new horizons.

## Figures and Tables

**Table 1:** Main titanium microstructural phases.

Phase	Symbol	Structure	Description	References
Alpha	α	HCP	Allotropic form of titanium at low temperature	
Beta	β	BCC	Allotropic form of titanium at high temperature	[56]
Alpha_2_	α_2_	HCP	A compound, Ti_3_Al, which appears in a wide range of Al content	[57]
Gamma	γ		The intermetallic compound TiAl	[58]
Alpha prime	α′	HCP	Martensitic metastable structure	[59]
Alpha double prime	A″	OR	Orthorhombic metastable martensitic structure	[60]
Beta prime or omega	β′ or ω	BCC	Metastable phases formed during quenching or aging	[61]

**Table 2:** List of the main titanium alloys that found application in the biological field, along with their most common microstructure, the specific applications that are commonly associated, and references to previous literature.

Alloy Name	Phase	Area of Application	References
CP-Ti (Grade 1)	α	Dental (uncommon)	[69,70]
CP-Ti (Grade 2)	α	Dental, Joint replacement	[71]
CP-Ti (Grade 3)	α	Dental	[72]
CP-Ti (Grade 4)	α	Joint replacement	[72]
Ti-8Al-1Mo-1V	α	-	[73]
Ti-6Al-2Nb-1Ta-0.8Mo	α	Joint replacement	[74,75]
Ti-6Al-2Zr-1Mo-1V	α	Joint replacement	[76]
Ti-6Al-4V (Grade 5)	α-β	Joint replacement, trauma, dental, spinal, etc.	[77]
Ti-6Al-4V ELI (Grade 23)	α-β	Joint replacement, trauma, cardiovascular, dental, spinal, etc.	[78]
Ti-6Al-7Nb	α-β	Joint replacement and dental	[79,80]
Ti-5Al-2.5Fe (Grade 9)	α-β	Dental	[81]
Ti3Al-2.5V	α-β	Joint replacement	[81]
Ti-6Al-6V-2Sn	α-β	Joint replacement	[82]
Ti-10Fe-10Ta-4Zr	α-β	Joint replacement	[83]
Ti-5Al-2Sn-2Zr-4Mo-4Cr	α-β	Joint replacement	[84]
Ti-4Al-4Mo-2Sn-0.5Si	α-β	Joint replacement	[85]
Ti-3Zr-2Sn-3Mo-25Nb	β	Joint replacement	[86]
Ti-13Nb-13Zr	β	Joint replacement and dental	[80]
Ti-12Mo-6Zr-2Fe	β	Joint replacement	[87,88,89]
Ti-15Mo	β	Joint replacement and dental	[90,91,92]
Ti-15Mo-5Zr-3Al	β	Joint replacement	[93,94]
Ti-15Mo-2.8Nb-0.2Si-0.260	β	Joint replacement	[92,95,96]
Ti-16Nb-10Hf	β	Joint replacement	[97,98]
Ti-35.5Nb-7.3Zr-5.7Ta	β	Joint replacement	[99,100]
Ti-29Nb-13Ta-4.6Zr	β	Joint replacement	[101,102,103]
Ti-24Nb-4Zr-8Sn	β	Joint replacement	[104]
Ti-9Mn	β	Joint replacement	[105,106]
Ti-6Mn-4Mo	β	Joint replacement	[106]
Ti-10Fe-10Ta-4Zr	β	Joint replacement	[107,108]
Ti-12Cr	β	Joint replacement	[109]
Ti-11Cr-0.xO	β	Joint replacement	[110]
Ti-36Nb-2Ta-3Zr-0.3O	β	Joint replacement	[111]
Ti-24Nb-0.5O	β	Joint replacement	[112]
Ti-24Nb-0.5N	β	Joint replacement	[112]
Ti-23Nb-0.7Ta-2Zr	β	Joint replacement	[113]
Ti-23Nb-0.7Ta-2Zr-1.2O	β	Joint replacement	[113,114]
Ti-12Mo-6Zr-2Fe	β	Joint replacement	[115]
NiTi (Nitinol)		Cardiovascular, dental, joint replacement	[116]
Ti-30Zr-xMo		Joint replacement	[117]

**Table 3:** Categories and biomedical applications for titanium alloys.

Category	Uses	Alloys	Ref.
Dental implants	Braces, bridges, abutments, orthodontics, fixation devices	β-titanium, pure titanium, Ti-6Al-4V, Nitinol	[187,188,189,190,191,192,193,194,195,196,197,198,199,200,201,202,203,204,205,206,207,208,209,210,211,212,213,214,215,216,217,218,219,220,221]
Orthopedic implants	joint components (stems, cups, …), meshes, bone substitutes, fixation devices	Ti-6Al-4V, Ti-6Al-7Nb, Ti-15Mo, Ti-13Nb-13Zr, pure titanium, Nitinol	[222,223,224,225,226,227,228,229,230,231,232,233,234,235,236,237,238,239,240,241,242,243,244,245,246,247,248,249,250]
Trauma devices	Plates, screws, rods, nails	Ti-6Al-4V, Ti-6Al-7Nb, and pure titanium	[251,252,253,254,255,256,257,258,259,260,261]
Spinal implants	cages, discs, fixation devices	Ti-6Al-4V, pure titanium	[262,263,264,265,266,267,268,269,270,271]
Cardiovascular devices	Heart valves, catheters, guidewires, clips, stents, implantable defibrillators, ventricular assist devices	Nitinol, Ti-6Al-4V, Ti-6Al-7Nb, Ti-15Mo, pure titanium	[272,273,274,275,276,277,278,279,280,281,282,283,284,285,286,287,288,289,290,291,292]
Soft tissue implants	Fixation devices, hernia meshes, breast reconstruction meshes	Ti-6Al-4V, Ti-6Al-7Nb, pure titanium	[293,294,295,296]

## Data Availability

Data sharing is not applicable.
